# OptNCMiner: a deep learning approach for the discovery of natural compounds modulating disease-specific multi-targets

**DOI:** 10.1186/s12859-022-04752-5

**Published:** 2022-06-07

**Authors:** Seo Hyun Shin, Seung Man Oh, Jung Han Yoon Park, Ki Won Lee, Hee Yang

**Affiliations:** 1 Department of Agricultural Biotechnology, Seoul National University, Seoul, 08826 Republic of Korea; 2 Bio-MAX Institute, Seoul National University, Seoul, 08826 Republic of Korea; 3 Research Institute of Agriculture and Life Sciences, Seoul National University, Seoul, 08826 Republic of Korea

**Keywords:** Natural compounds, Chemical-protein interaction, Multi-target prediction, Deep learning, Siamese neural network

## Abstract

**Background:**

Due to their diverse bioactivity, natural product (NP)s have been developed as commercial products in the pharmaceutical, food and cosmetic sectors as natural compound (NC)s and in the form of extracts. Following administration, NCs typically interact with multiple target proteins to elicit their effects. Various machine learning models have been developed to predict multi-target modulating NCs with desired physiological effects. However, due to deficiencies with existing chemical-protein interaction datasets, which are mostly single-labeled and limited, the existing models struggle to predict new chemical-protein interactions. New techniques are needed to overcome these limitations.

**Results:**

We propose a novel NC discovery model called OptNCMiner that offers various advantages. The model is trained via end-to-end learning with a feature extraction step implemented, and it predicts multi-target modulating NCs through multi-label learning. In addition, it offers a few-shot learning approach to predict NC-protein interactions using a small training dataset. OptNCMiner achieved better prediction performance in terms of recall than conventional classification models. It was tested for the prediction of NC-protein interactions using small datasets and for a use case scenario to identify multi-target modulating NCs for type 2 diabetes mellitus complications.

**Conclusions:**

OptNCMiner identifies NCs that modulate multiple target proteins, which facilitates the discovery and the understanding of biological activity of novel NCs with desirable health benefits.

**Supplementary Information:**

The online version contains supplementary material available at 10.1186/s12859-022-04752-5.

## Background

Natural product (NP)s, are defined as substances produced by living organisms. The term NP is often used broadly, covering both natural compound (NC)s and mixtures of NCs derived from natural sources [1]. The diverse biological activities of NPs are due to the activity of their constituent NCs. In order to fully understand and utilize NPs, it is important to identify and investigate the mode of action of active NCs.

Subject to selective pressure over millions of years, NCs now have diverse bioactive chemotypes, some of which are optimized for particular biological functions, such as endogenous growth and the defense of living organisms [2]. Due to their wide range of target space and bioactivity, NCs have been an important foundation for traditional medicines and modern pharmacology. The World Health Organization has reported that approximately 20,000 plants are used for herbal remedies in 91 countries worldwide [3]. Diverse bioactive scaffolds of NCs have been examined as drug candidates for the treatment of various diseases, including cancer, cardiovascular diseases, and infectious diseases [2]. From 1981 to 2014, more than a half of all approved novel drugs were derived from NCs [4]. In addition to pharmaceutical sources, NC applications are also expanding in the functional food, cosmetics and agricultural industries [5–8].

Another interesting characteristic of NCs is their propensity to modulate multiple protein targets. The unique scaffolds and structural motifs of NCs enable interactions with multiple target proteins to elicit diverse biological activities [9]. In the past few decades, the paradigm of drug discovery has shifted from 'one target, one disease’ to ‘multi-target’ or ‘multimodal’ drug discovery, especially for diseases with complex etiologies or drug resistance issues [10]. Such multi-target modulating drugs have been expected to improve safety issues and enhance clinical efficacy compared to single-target drugs [11–13]. For example, resveratrol, a well-known stilbenoid found in red wine and various foods, has been associated with 21 direct molecular targets including SIRT1 [14]. Resveratrol has been shown in clinical studies to have beneficial effects on pathways implicated in a variety of diseases, such as diabetes, obesity, various types of cancer, Alzheimer's disease and cardiovascular disease [15]. However, due to the NCs targeting multiple proteins, it has been challenging to discover optimal NCs, which regulate desired targets and avoid any off-targets.

Various experimental methods have been developed over the past few decades to identify NCs that regulate target proteins [2]. However, the discovery process for multi-target modulating NCs is tedious and cost-intensive. For translation of discoveries into drug development, a pre-approval process of lead identification, compound optimization, in vitro and animal experiments is required before the first clinical trial can be conducted. Developing a new drug through this process typically costs in excess of 800 million US dollars [16]. In recent years, computational methods have provided substantial assistance in the discovery of new NCs. Molecular descriptors and fingerprint methods have made it possible to describe NCs in mathematical expressions [17], while 3D modeling and docking methods have been developed to simulate a complex molecular structure and conformational space of NCs, as well as their interactions with target proteins [18]. These cheminformatics tools, along with chemical-protein interaction databases, have shed light on the development of machine learning models that predict novel NCs and streamline the NC discovery process.

A variety of machine learning methods have been trained on chemical-protein interaction databases to predict compounds with novel target modulating activities [19–21]. Of particular note, deep neural networks (DNN)s have been widely applied in the field of active compound discovery, as they enable the automation of the feature engineering process that often becomes a bottleneck in conventional machine learning methods. DNN’s capabilities with end-to-end learning—a process of training parameters jointly—enables the automation of complicated predictive procedures ranging from data pre-processing to the prediction of processed data. Thus, DNN efficiently generates or extracts important hidden features from the input vectors of the compounds responsible for their activities, circumventing the need for manually engineering the input features [22]. However, conventional machine learning models including DNN still carry limitation: their performance greatly relies on the data quantity and quality [23, 24]. Unfortunately, established chemical-protein interaction databases are biased towards the most well-studied proteins and compounds. Thus, not all proteins have sufficient data to reliably train machine learning models. In addition, most existing databases are optimized for binary classification methods as they only provide single or limited target protein data for each compound. Datasets from such databases treat unknown chemical-protein interactions as negative data. Consequently, predictive models trained on single-label data run the risk of predicting false-negative interactions when the interactions can be experimentally tested positive. Thus, the prediction of such interactions should be treated as a multi-label classification task. Considering that the interaction data provided by the existing databases is single-labeled and limited in size, a model capable of learning multi-classification from single labeled data is required to overcome the current limitations of NC discovery.

Siamese neural network (SNN), first suggested in the early 1990s by Bromley and LeCun, is comprised of two identical networks, with one called ‘heads’, that accepts distinct input pairs and is an activation function called 'body', that concatenates the two heads [25]. SNN is a powerful tool for two reasons: it enables similarity comparisons of complex data and can be applied to one-shot and few-shot learning. When comparing the similarity between two high-dimensional data points, the SNN learns the hidden representations of the two input vectors in a parallel fashion and compares the outputs at the end using a similarity metric such as cosine distance. Unlike models that use classification loss functions to classify between classes, SNNs use contrastive loss functions to learn to distinguish between inputs. The procedure of SNN to generate pairs of similar and dissimilar data points from the original data for training allows SNN to train on larger data compared to the original dataset. In addition, SNN is a model capable of few-shot learning, a learning method that can make predictions using a single or a very small number of samples [26]. Also, since the model is trained to predict similarity between input pairs, the performance of the model is not impacted by the class imbalance in positive and negative data. Due to these characteristics of SNN, it is a significantly compatible model, especially for learning protein-interacting compound data. SNN has been applied to various fields including image analysis, audio and speech processing, and sensor-based activity recognition [27–30]. Furthermore, SNN has also been recently applied in the field of pharmacology. ReSimNet, a model for drug discovery and repositioning was developed by Jeon et al. in 2019 [31]. Jeon and colleagues used SNN to predict transcriptional response similarities between two compounds using gene expression data from the CMap database. However, there has not yet been a case where an SNN has been applied to predict NCs that modulate multiple disease-specific target proteins.

Here, we describe ‘OptNCMiner’, a machine learning model suitable for predicting ‘optimal NCs’ that modulate disease-specific multi-targets. Built on a structure of SNN, OptNCMiner preserves the advantages of DNN to effectively extract essential features of NCs related to chemical-protein interactions. OptNCMiner is validated on its ability with multi-label learning from single positive data on chemical-protein interactions, and is also capable of few-shot learning, enabling multi-class classification on NCs using small datasets. We tested OptNCMiner with the discovery of natural sources containing NCs that regulate target proteins associated with type 2 diabetes mellitus (T2DM)-related complications.

## Methods

**Fig. 1:**
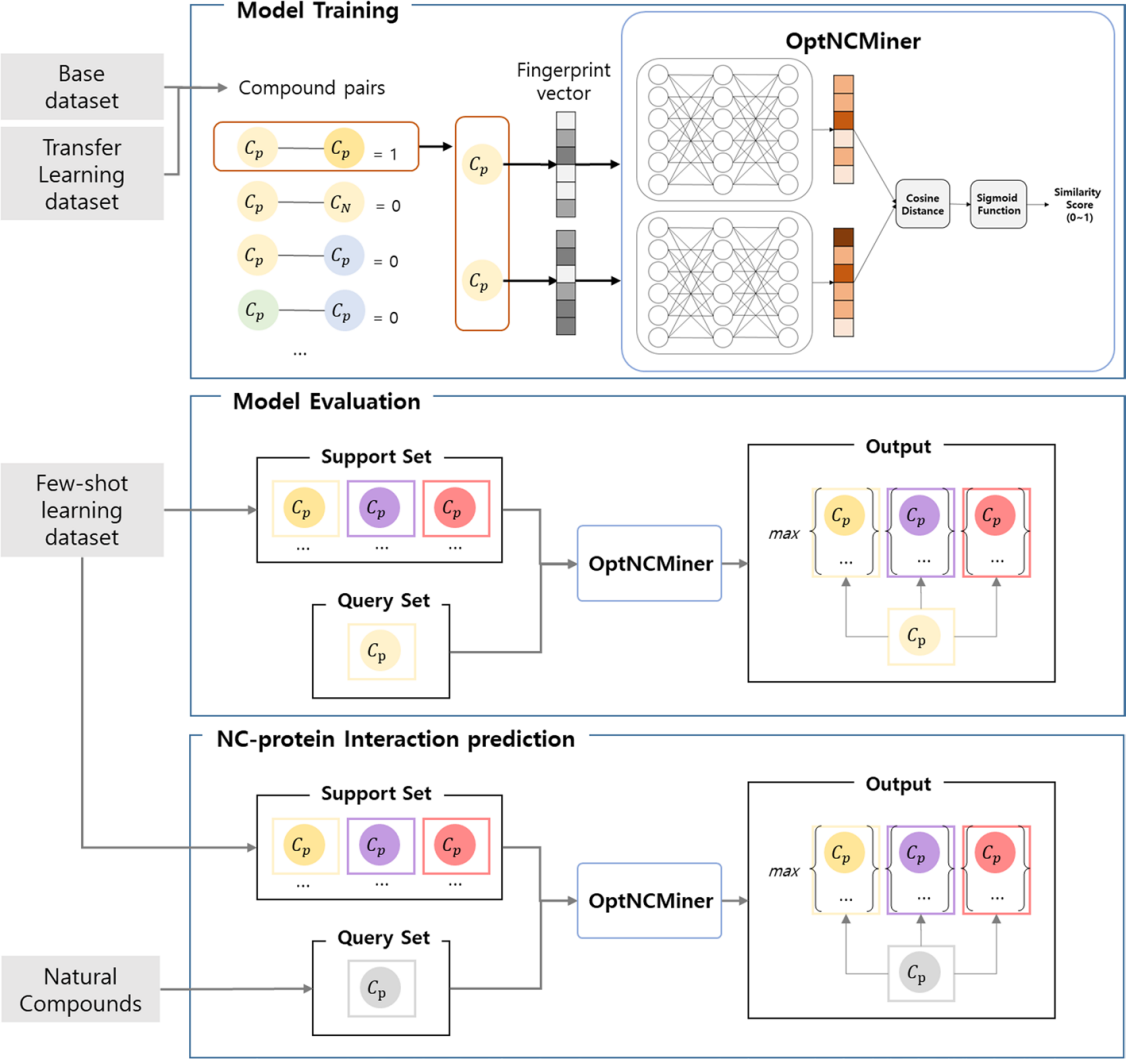
OptNCMiner model flowchart

OptNCMiner learns structural similarities between compound pairs and is trained to grant high similarity scores to chemical pairs where both compounds are active against a single target protein. Upon successful training, OptNCMiner calculates the activity score of NCs with each target protein in of the context of a similarity score between NCs and known active compounds of target proteins (Fig. 1). OptNCMiner was trained with three datasets of different sizes in order to test its learning capability regardless of dataset size. Comparisons with traditional classification models and validation of false positives using in silico docking simulation revealed that OptNCMiner successfully predicts both known and unknown chemical-protein interactions.

### Data collection and preparation

Chemical-protein interaction data was gathered from ExCAPE-DB and LIT-PCBA. ExCAPE-DB is a database of chemogenomics data curated from two major public databases: PubChem and ChEMBL [32]. Compound data curated from ExCAPE-DB were labeled according to the activity flag provided by ExCAPE-DB: ‘A’ for active and ‘N’ for inactive. LIT-PCBA is a dataset designed for virtual screening and machine learning based on PubChem bioassay data [33]. Similarly, compound data curated from LIT-PCBA were labeled as active or inactive based on the given activity class for each compound.

Three datasets of different sizes were prepared and named as the 'base dataset', 'transfer learning dataset’, and 'few-shot learning dataset’ in accordance with their decreasing size (Table 1). All datasets were composed of active and inactive compounds. The base dataset was constructed with chemical-protein interaction data for 11 proteins and data for more than 5,000 active compounds, randomly selected from ExCAPE-DB. The transfer learning dataset was constructed with chemical-protein interaction data for 7 proteins with the number of actives between 500 and 1,000. The transfer learning dataset was used for transfer learning of OptNCMiner and five baseline models capable of multi-class classification: Cosine similarity, Naïve Bayes (NB), Logistic Regression (LR), Random Forest (RF), and Multi-layer Perceptron (MLP). Data for the transfer learning dataset were collected from ExCAPE-DB and LIT-PCBA. The performance of OptNCMiner was compared to that of the baseline models. The few-shot learning dataset was constructed with chemical-protein interaction data for 7 proteins with data for less than 100 active compounds. The few-shot learning dataset was used for the few-shot learning of OptNCMiner and was collected from LIT-PCBA data. This set consists of 296 compounds in total.

**Table 1:** Data used to construct the base dataset, transfer learning dataset, and few-shot learning dataset

Dataset	Target gene	Target protein	Data source	Active compounds	Inactive compounds
Base dataset (actives $>$ 5000)	ADORA2A	Adenosine receptor A2a	ExCAPE-DB	5077	591
	BRCA1	Breast cancer type 1 susceptibility protein	ExCAPE-DB	8619	43,095
	CNR1	Cannabinoid receptor 1	ExCAPE-DB	5125	397
	DRD2	D(2) dopamine receptor	ExCAPE-DB	8037	40,185
	HTR1A	5-hydroxytryptamine receptor 1A	ExCAPE-DB	6339	31,695
	KCNH2	Potassium voltage-gated channel subfamily H member 2	ExCAPE-DB	5327	26,635
	LMNA	Prelamin-A/C	ExCAPE-DB	14,533	72,665
	OPRM1	Mu-type opioid receptor	ExCAPE-DB	5665	2872
	SLC6A4	Sodium-dependent serotonin transporter	ExCAPE-DB	6912	370
	TARDBP	TAR DNA-binding protein 43	ExCAPE-DB	12,193	60,965
	TDP1	Tyrosyl-DNA phosphodiesterase 1	ExCAPE-DB	23,129	115,645
Transfer learning dataset (1000 > actives > 500)	ADRA2A	Alpha-2A adrenergic receptor	ExCAPE-DB	816	39
	GRIN1	Glutamate receptor ionotropic	ExCAPE-DB	553	92
	HTR3A	5-hydroxytryptamine receptor 3A	ExCAPE-DB	565	65
	MINK1	Misshapen-like kinase 1	ExCAPE-DB	929	8
	PKM2	Pyruvate kinase PKM	ExCAPE-DB	546	2730
	POLK	DNA polymerase kappa	LIT-PCBA	772	3860
	VDR	Vitamin D3 receptor	LIT-PCBA	884	4420
Few-shot learning dataset (100 > actives)	ADRB2	Beta 2 adrenergic receptor	LIT-PCBA	17	170
	ESR	Estrogen receptor alpha	LIT-PCBA	13	130
	IDH1	Isocitrate dehydrogenase	LIT-PCBA	39	390
	MTOR	mammalian target of rapamycin complex 1	LIT-PCBA	97	970
	OPRK1	Kappa opioid receptor	LIT-PCBA	24	5460
	PPARG	Peroxisome proliferator-activated receptor gamma	LIT-PCBA	27	270
	TP53	Cellular tumor antigen p53	LIT-PCBA	79	790

### Input generation

For each compound, a standard fingerprint of 1024 bits was generated from the canonical SMILES representation using Chemistry Development Kit (CDK) in R software [34]. For each of the base dataset and transfer dataset, 10% of the data were set aside for the test set. Of the remaining 90%, 10% were set aside for use as the validation sets. As a result, 81% of the total data were used for training, 9% for validation, and 10% for testing.

We have generated pairs of compounds and their labels, since OptNCMiner is a network that accepts inputs in the form of pairs and computes the similarity between the two. Compound pairs were labeled as ‘positive’ if both were classified as active for the same target protein. Those pairs that did not satisfy the criteria were labeled as ‘negative’. In order to prevent proteins with large interaction data sizes from dominating the training dataset, proteins were randomly sampled from a uniform distribution. For generating positive pairs, active compounds to the protein, which is shown as ${C}_{p}$ in Fig. 1, were randomly sampled. On the other hand, compounds interacting with different proteins or negative compounds to the target protein (${C}_{N}$ in Fig. 1) were randomly chosen to generate negative pairs. 7,000 positive and negative pairs each were generated for the training dataset, in the form of fingerprint vectors of compound pairs concatenated with the binary labels of either 1 or 0. This add up to 14,000 pairs generated from base dataset for pre-training the model. Previous studies by others have successfully predicted the properties of chemicals using pre-training data with about 20,000 chemicals or less [35, 36]. So, we decided that our pre-training data was a reasonable size to train the model without undue computational cost.

### Model building

OptNCMiner is built in an SNN structure, where pairs of inputs are fed to identical multi-layer perceptrons called ‘head function’ to generate pairs of embedding vectors. The similarity between two embedding vectors is computed by a distance function referred to as the ‘body function’. The overall structure of the model is represented as follows:

1 $$Y=B(H\left({X}_{1}\right), H\left({X}_{2}\right))$$

where ${X}_{1}$, ${X}_{2}$ is a pair of chemical inputs in the form of fingerprint vectors, Y is the binary label of the pair, H(·) is the head function, and B(·) is the body function. The head function H(·) maps input vectors ${X}_{1}$, ${X}_{2}\in {R}^{2048}$ into embedding vectors ${Z}_{1}$, ${Z}_{2}\in {R}^{2048}$. The hidden layer dimension for H(·) is set to 2048–2048–2048 with a dropout of 0.5, and was constructed with PyTorch. The resulting embedding vectors are created in forms of ${Z}_{1}={W}_{2}f({W}_{1}{X}_{1}+{b}_{1}+{b}_{2})$ and ${Z}_{2}={W}_{2}f({W}_{1}{X}_{2}+{b}_{1}+{b}_{2})$, where f(·) is a ReLU activation function and ${W}_{1}\in {R}^{h\times 2048}, {W}_{2}\in {R}^{e\times h}, {b}_{1}\in {R}^{h}, {b}_{2}\in {R}^{e}$ are trainable weights and biases, respectively. ${Z}_{1}$ and ${Z}_{2}$ are then fed in to the body function B(·), which is a function of cosine distance, defined by Eq. (2). We then defined the structural similarity of two compounds as the cosine distance between two embedding vectors. Finally, sigmoid function with Eq. (3) is used to produce the output called 'similarity score', where 1 refers to the presence of chemical-protein interaction, and 0 refers to its absence.

2 $$cosine \,distance\left({Z}_{1},{Z}_{2}\right)=\frac{{Z}_{1}\bullet {Z}_{2}}{\Vert {Z}_{1}\Vert \times \Vert {Z}_{2}\Vert }=\frac{\sum_{i=1}^{n}{{Z}_{1}}_{i}\times {{Z}_{2}}_{i}}{\sqrt{\sum_{i=1}^{n}{{{Z}_{1}}_{i}}^{2}}\times \sqrt{\sum_{i=1}^{n}{{{Z}_{2}}_{i}}^{2}}}$$

3 $$\upsigma \left(\mathrm{x}\right)= \frac{1}{1+{e}^{-x}}$$

Binary Cross Entropy (BCE) was used as the loss function (4). Through the optimization process, the model was trained to minimize BCE between the predicted output and the label. The model hyperparameters were optimized using the validation set during training. We used Adam optimizer [37] with a learning rate of 0.0001.

4 $$\mathrm{BCE}= -\frac{1}{N}\sum_{i=0}^{N}{y}_{i}\bullet \mathrm{log}\left({\widehat{y}}_{i}\right)+(1-{y}_{i})\bullet \mathrm{log}(1-{\widehat{y}}_{i})$$

The training data were utilized in the support set upon completion of training. A support set was constructed by randomly sampling 100 active compounds per protein. Each query in the test set was compared to each compound in the support set. To classify the binding of the compound to the target protein, the highest similarity for each protein was compared to a threshold. Then, for each protein, the maximum similarity score of the query compound is used to classify whether the compound binds to the protein. A threshold of 0.5 is used to determine the binding of the compound to the target protein.

### Training approaches for varying dataset sizes: transfer learning and few-shot learning

For many machine learning problems, better performance can be achieved by applying a transfer learning method, which pre-trains the model on a larger dataset before further training it on the target dataset [38]. We previously constructed the transfer learning dataset, which is composed of chemical-protein interaction data smaller in size (500 ~ 1000 active compounds) than the base dataset. We have adopted the idea of transfer learning on the transfer learning dataset, by pre-training the model with the base datasets and fine-tuning it with the transfer learning dataset.

For datasets too small to feasibly train on, few-shot learning can be used. The few-shot learning method uses distance metrics to compute the similarity between a data point in a support set and a data point in a query set [26]. In this study, we used the cosine distance to compute the similarity between the embedding vectors of a query data point and a support data point. If the distance between a query data point and at least one support data point is greater than 0.5, the query data point was predicted to regulate the target protein, and the query data point received a positive label for the target protein. We used the model trained for transfer learning to predict chemical-protein interactions with the few-shot learning dataset, taking the sparse available data and using it as a support set. 90% of the available data for the few-shot learning dataset was used as support and was tested on the remaining 10%.

### Multi-label classification from single label data

Chu and colleagues have provided an updated gold standard dataset of chemical-protein interaction data that accounts for multiple bindings [39]. To test the viability of learning multi-label representation from single-label data, the performance of OptNCMiner learning from a filtered single-label version of this data tested on the single-label test data was compared to the testing performance of the full multi-label data. performance with both single-label and multi-label datasets is shown in Additional file 1: Table S1 and all datasets used for the trial are available at the GitHub address listed in ‘Availability of data and materials’ section.

### Model evaluation metrics

Since OptNCMiner is a classification model, typical classification metrics have been used to evaluate the performance of the model. A recall is a metric that measures the proportion of true positives, ${T}_{p}$, against all existing positives (5). Thus, recall is a metric that evaluates misclassification of actual positives. Accuracy measures the general performance of the model, by calculating the proportion that the model classifies correctly among the entirety of the predictions (6). The area under the receiver operating characteristics (AUROC) is an area under the curve drawn on the plot between the true positive rate (TPR) and false positive rate (FPR). The AUROC value represents the degree of class separability of the model. The final evaluation metrics are calculated as the weighted average of metric values of all proteins.

5 $$\mathrm{Recall}= \frac{{T}_{p}}{{T}_{P}+{F}_{N}}$$

6 $$\mathrm{Accuracy}= \frac{{T}_{p}+{F}_{P}}{{T}_{P}+{T}_{N}+{F}_{P}+{F}_{N}}$$

## Results and discussion

### Dataset analysis

The chemical-protein interaction data from three differently-sized datasets are aggregated into a total of 106,317 positive interactions with 25 different target proteins. Since all proteins have different instances of interactions, 1,000 active compounds were randomly selected for each protein in the base dataset, and all active compound data from the transfer learning data and few-shot learning data were used to investigate the target protein-interacting compound space.

**Fig. 2:**
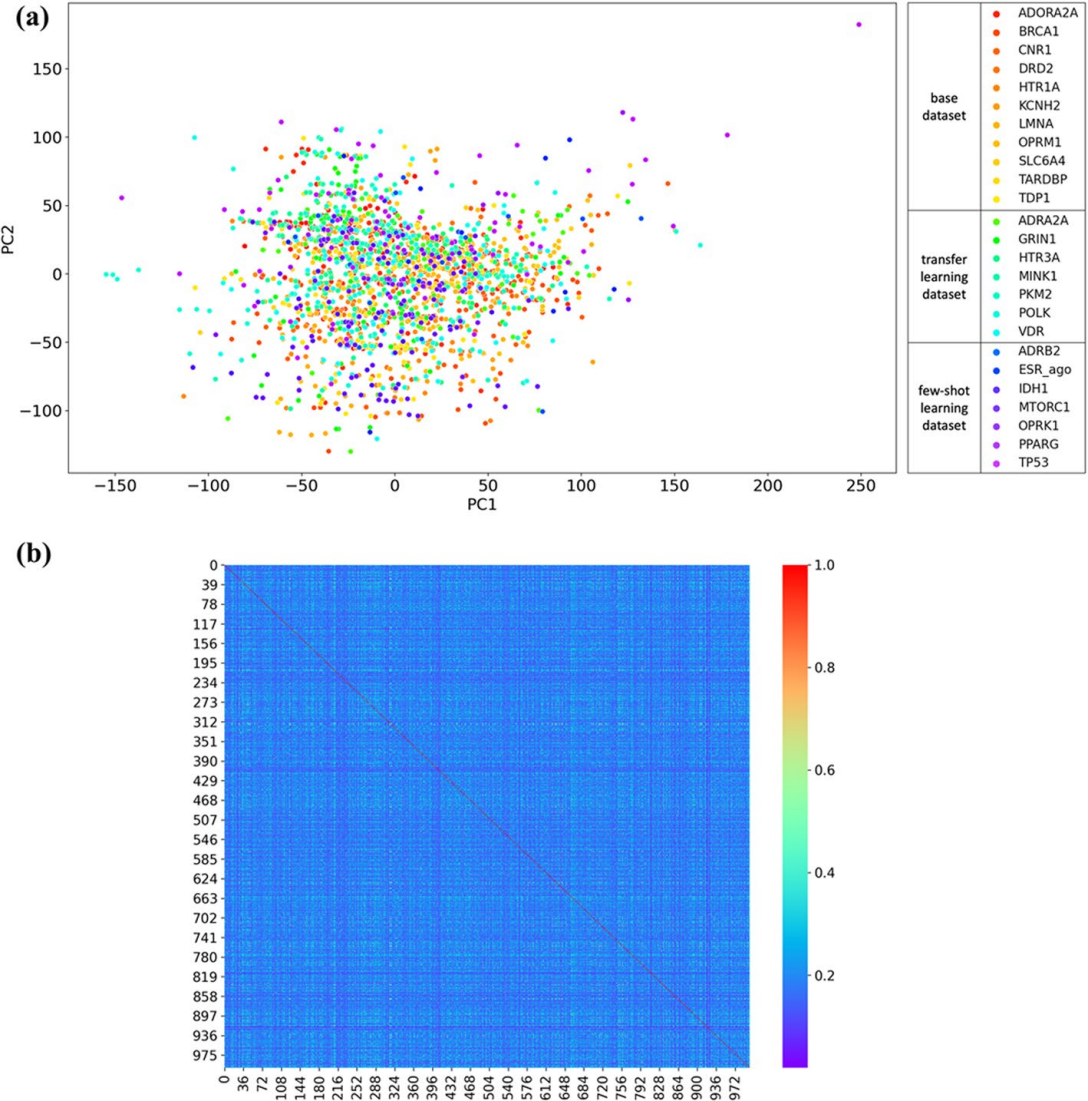
The distribution of **a** physicochemical properties; and **b** chemical structures in the base dataset, transfer learning dataset, and few-shot learning dataset

To explore the physicochemical properties of the sampled compounds, principle component analysis (PCA) was used. The following ten physicochemical properties were calculated using OPERA 2.6 [40] to generate the PCA plot: Octanol–water partition coefficient (LogP), melting point (MP), boiling point (BP), vapor pressure (VP), water solubility (WS), Henry’s Law constant (HL), Octanol–air partition coefficient (KOA), retention time (RT), acid dissociation constant (pKa), and pH-dependent lipid-aqueous partition coefficient (LogD). The scattered compounds are represented (in Fig. 2a) as a 2D diagram of PCA, where the compounds are assigned with different color codes according to the proteins they interact with.

Additionally, the structural similarity distribution of the sampled compounds was investigated using a pairwise Tanimoto similarity matrix. Sampled compounds were represented in a high dimensional space with a 1,024-bit standard fingerprint, containing information for the chemical substructures. All possible pairs of compounds were generated from those sampled and Tanimoto similarity values between 1,024-bit standard fingerprint vectors of chemical pairs were calculated. The resultant similarity matrix was rendered in the form of a heat map (Fig. 2b), where the sampled compounds were positioned in an order of base dataset, a transfer learning dataset, and a few-shot learning dataset.

The PCA plot in Fig. 2a reveals that compounds interacting with 25 different proteins share similar physicochemical properties and cannot easily diverge. However, the Tanimoto similarity values calculated from the fingerprint vectors of the compound pairs are distributed mostly between 0.1 to 0.3, which means almost all of the sampled compounds are structurally different from each other. From Fig. 2a, b, we can conclude that our compounds share similar physicochemical properties but are structurally diverse. Thus, there was no predetermined structures or physicochemical properties that facilitated the prediction of the different target proteins. Using such compound data, OptNCMiner was trained to learn these hidden features to discern the binding natures of the compounds with different target proteins.

### Performance evaluation

To evaluate whether OptNCMiner is sufficiently specialized for NC multi-target prediction, we examined its ability to learn multi-label classification by comparing the performance of the model after training with single-label and multi-label data. The single-label data were generated by deleting classes—in this case, target proteins of compounds from the original multi-label data. In a manner similar to how Cole validated multi-label learning with single-label data, we show that OptNCMiner has the ability to identify multiple targets for compounds from single-label data [41]. OptNCMiner achieved similar recall and AUROC values when trained on single-label and multi-label data and then tested with the multi-label test dataset (Additional file 1: Table S1).

The performance of OptNCMiner was also evaluated with the compounds not used in the training pair generation from the base dataset and transfer dataset. Our framework allows for the prediction of multiple binding targets of NCs not included in the original binding data; since the test data is labeled in a single-positive manner, the predicted output contains a high number of false positives. Therefore, recall (the ratio of predicted positives among the entire positives) is considered to be the most relevant evaluation metric.

The performance of OptNCMiner was compared with five baseline models capable of multi-label classification, which are cosine similarity, NB, LR, RF, and MLP. The baseline models were trained with the same training set and were evaluated using the same test set to that of OptNCMiner, generated from the base and transfer learning datasets. For the cosine similarity method, we calculated the cosine similarity of two standard fingerprint vectors. If the cosine similarity value of a pair exceeded 0.5, two compounds were considered structurally similar and the test compound is predicted to bind to the target protein, and vice versa. The remaining baseline models were trained to classify pairs of compounds with binary labels of 1 or 0. Binary label of 1 indicates that the test chemical is expected to bind to the same target protein because it is similar to its pair. The performance of the baseline models was evaluated with the base dataset and transfer learning dataset. All baseline models were first trained and evaluated with the base dataset, while MLP was further trained in the manner of the transfer learning with the transfer learning dataset. As transfer learning is not applicable to cosine similarity, NB, LR, and RF, the performance of these models was simply evaluated after training on the transfer learning dataset.

Table 2 shows that OptNCMiner outperformed the baseline models. The recall values of OptNCMiner were 0.833 and 0.871 for the base and transfer learning datasets, respectively. These results indicate more than 80% of the known positives were correctly predicted. The second best recall values were found with RF of 0.677 for the base data set and MLP of 0.824 for the transfer learning data set. Some baseline models achieved recall values lower than 0.5, which means the models predicted less than half of the positive interactions accurately: NB, LR, and MLP for the base dataset and NB and RF for the transfer learning dataset. The values of AUROC for OptNCMiner on both the base dataset and transfer learning dataset were above 50%, demonstrating that the model’s discriminant power is better than random chance. The relatively low accuracy and AUROC values may be due to newly predicted compound targets, which result in high false positive rates. Cosine similarity, NB, LR, and MLP generated accuracy values above 0.7, but AUROC values were relatively low, suggesting that the methods are vulnerable to data imbalances. All evaluation metric values have been improved in the transfer learning dataset compared to the base dataset for OptNCMiner. Considering that both the base dataset and the transfer learning dataset held diverse compound structures, the improved evaluation metrics denote that the performance of the model was improved by the transfer learning method, regardless of the data characteristics. MLP, a model capable of transfer learning in addition to OptNCMiner, also showed improved recall and AUROC values after transfer learning. However, in recall, the most important performance metric, OptNCMiner was superior for both the base dataset trained model and the transfer learning dataset trained model.

**Table 2:** The performance of OptNCMiner and baseline models with the base dataset and transfer learning dataset

Model	Performance metric^1^	Base dataset	Transfer learning dataset
OptNCMiner	Recall	0.833	0.871
	AUROC	0.632	0.787
	Accuracy	0.440	0.713
Cosine similarity	Recall	0.573	0.696
	AUROC	0.643	0.761
	Accuracy	0.708	0.818
Naïve bayes classifier	Recall	0.322	0.483
	AUROC	0.623	0.696
	Accuracy	0.909	0.887
Logistic regression	Recall	0.212	0.581
	AUROC	0.606	0.785
	Accuracy	0.978	0.969
Random forest	Recall	0.677	0.479
	AUROC	0.343	0.241
	Accuracy	0.028	0.027
Multi-layer perceptron	Recall	0.361	0.824
	AUROC	0.676	0.818
	Accuracy	0.972	0.899

^1^All performance metrics are weighted averages of the results of all proteins comprising the dataset

Another strong advantage of OptNCMiner is its ability to predict chemical-protein interactions for proteins with limited training data. The trained and transfer-learned OptNCMiner using the base dataset and transfer learning dataset was used to predict chemical-protein interactions in the few-shot learning dataset using a few-shot learning method. The few-shot learning performance of OptNCMiner was evaluated with 7 proteins with data for less than 100 interacting compounds and is demonstrated in Table 3. OptNCMiner achieved 0.829 for the weighted average of recall and 0.665 for the weighted AUROC average. Although Beta 2 adrenergic receptor and Isocitrate dehydrogenase show relatively poor performance, the model’s predictions are remarkably accurate for most proteins. The performance is not necessarily correlated to the number of available data points, which is shown in the last column of Table 3, indicating that either the presence of some interactions were not identified by the network, or is indicative of a lack of representation present in the small data. Thus, it is confirmed that OptNCMiner possesses the ability to identify structural properties of compounds that enable specific chemical-protein interactions from a small number of samples.

**Table 3:** Performance of OptNCMiner with the few-shot learning dataset

Target protein	Recall	AUROC	Accuracy	Count
Beta 2 adrenergic receptor	0.488	0.400	0.450	5
Estrogen receptor a	0.585	1.000	0.764	5
Isocitrate dehydrogenase	0.488	0.600	0.536	5
Mammalian target of rapamycin complex 1	0.537	0.889	0.663	9
Kappa opioid receptor	0.659	1.000	0.806	5
Peroxisome proliferator-activated receptor gamma	0.610	1.000	0.778	5
Cellular tumor antigen p53	0.537	0.857	0.664	7
Weighted average	0.555	0.829	0.665	41

### Output validation

OptNCMiner is a model capable of predicting multiple target proteins for NCs through multi-label learning. OptNCMiner predicts not only the known protein targets of NCs, but also unknown target proteins, which results in high false positive rates. To confirm OptNCMiner’s ability to predict unknown chemical-protein interactions, we sought to validate OptNCMiner’s false positive outputs in few-shot learning.

**Fig. 3:**
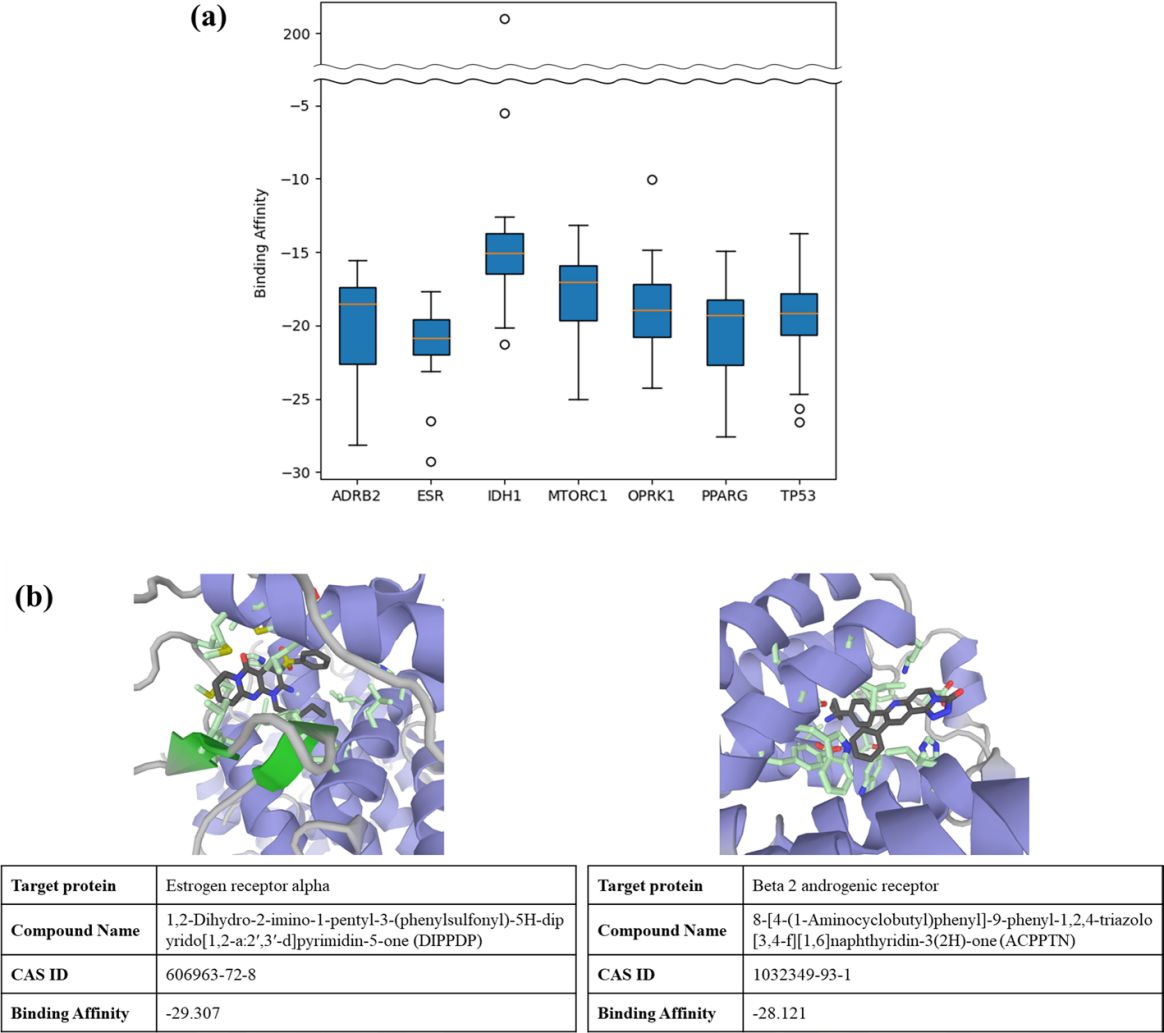
In silico docking score for false positives from the **a** few-shot learning dataset; and **b** two molecular docking results for compound-protein interactions with lowest in silico docking score (highest binding affinity)

OptNCMiner went through few-shot learning using the few-shot learning dataset, where chemical-protein interactions were predicted among 7 proteins and 23 compounds in the test set. To examine whether the false positives were real negatives or newly discovered target proteins, literature searches and in silico docking using GalaxyDockWEB [42] were undertaken. Among the 115 false positives, 4 chemical-protein interactions were validated in the literature (Additional file 1: Table S2). All 115 compounds went through in silico docking. As shown in Fig. 3a, 114 of the 115 chemical-protein interactions generated a negative binding affinity score (see Additional file 2), suggesting that binding of the ligand to the active site exists in a favorable energy state [43]. The result of in silico docking indicates that the false positives predicted by OptNCMiner are real unknown positives with a high probability. Two examples of successful docking of compounds and target proteins, where chemical-protein interactions were previously unknown, are illustrated in Fig. 3b. The examples were selected based on the two lowest binding affinities among the 115 false positives, which were − 29.307 and − 28.121 respectively. In both examples, the compounds DIPPDP and ACPPTN (dark grey), are stably docked in the pre-assigned binding pockets with interacting amino acid side chains of the target proteins, estrogen receptor alpha and beta 2 androgenic receptor (light green).

### Use case scenario: NCs present in natural sources that modulate target proteins associated with T2DM complications

To investigate the practical application of OptNCMiner in novel NC discovery, the program was trained using the base dataset to identify NCs present in natural sources that modulate target proteins associated with T2DM complications. Diabetic nephropathy, diabetic keratopathy, and cardiomyopathy were chosen as T2DM complications and target proteins for each complication were identified based on previous reports [44]. Among the identified target proteins, 8 proteins with interacting compound data were selected and used as target protein candidates: Peroxisome proliferator activated receptor α (PPARα), Yes-associated protein (YAP), Phosphoinositide 3-kinase (PI3K), Protein kinase C β (PKCβ), Toll-like receptor 4 (TLR4), Sodium/glucose cotransporter 2 (SGLT2), G protein-coupled receptor 120 (GPR120), and Nuclear factor erythroid 2-related factor 2 (Nrf-2) (Additional file 1: Table S3). The interacting compound data was gathered from BindingDB, a chemical-protein interaction database [45]. The sizes of interacting compound data varied among the target proteins. Transfer learning or few-shot learning was applied according to the two different ranges of data size. Five target proteins with more than 100 interacting compound data points were assigned to transfer learning and three target proteins with less data were assigned to few-shot learning. In order to predict NCs in natural sources, data including NCs and natural resources containing NCs were obtained from FooDB, a database of food constituents [46]. First, the pre-trained OptNCMiner using the base dataset was transfer-learned based on chemical-protein interaction data for the target proteins assigned. Second, 65,038 NCs from FooDB were then provided as input to the transfer-learned OptNCMiner to predict protein targets. Third, the transfer-learned OptNCMiner was used for few-shot learning of three target proteins assigned for few-shot learning. In the same manner, NCs from FooDB were provided as input to predict chemical-protein interactions for the three proteins. OptNCMiner achieved a high recall value for all proteins (Additional file 1: Table S4).

**Fig. 4:**
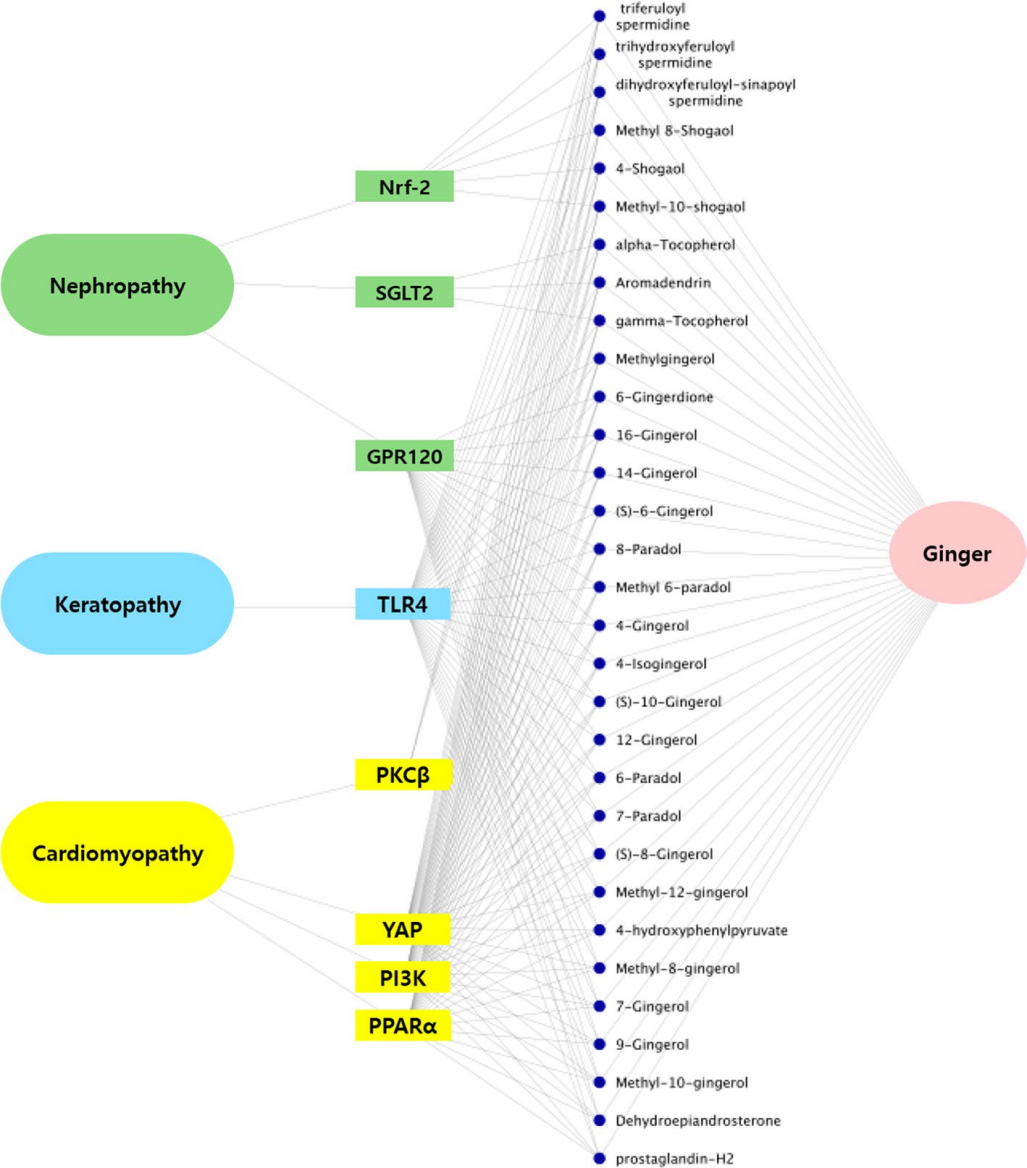
OptNCMiner predicts that ginger contains 33 NCs that regulate 8 different target proteins associated with T2DM complications

To visualize the network of relationships between T2DM complications and NCs of food origin (Fig. 4), a standardized process was followed. First, chemical-protein pairs with a score above 0.5 were selected. Of these pairs, NCs with the highest number of target proteins were selected. Here, 102 NCs modulating 5 different target proteins were chosen. The 102 NCs were matched with their food sources, which added up to 680, as most NCs are found in multiple food sources (see Additional file 3). For ease of visualization, a food source that contains the highest number of selected NCs is shown in Fig. 4. The relationships between T2DM complications, target proteins, NCs, and foods were visualized using connected edges. In identifying food sources to fight T2DM complications, the figure shows that ginger contains 31 NCs that modulates 8 different target proteins associated with T2DM complications.

Ginger is a herbaceous plant that has a long history of use in traditional medicines and foods. The root of the plant contains a vast array of NCs which are responsible for a wide range of biological activities, including anti-diabetic effects, gastrointestinal protection, anti-cancer effects, cardiovascular protection, and the prevention of obesity [47, 48]. Among the 160 identified NCs present within ginger, phenolic and terpene compounds, such as gingerols and shogaols, have been widely investigated for their pungent stringency, abundance, and multiple health benefits [47, 49, 50]. Derivatives of gingerols and shogaols represented a large proportion of ginger-derived NCs identified by OptNCMiner. Interestingly, OptNCMiner predicted 6-gingerol as an NC that ameliorates T2DM complications, by modulating 5 different proteins: TLR4, PI3K, YAP, PPARα, and GPR120.

TLR4 is a receptor protein with multiple physiological functions, and has been implicated in the weakening of ocular surface and corneal nerves, leading to diabetic keratopathy. TLR4 binds to high-mobility group box 1 protein that activates the NF-κB pathway, leading to inflammation in the cornea [51]. Interestingly, it has been reported that gingerols including 6-gingerol and 6-shogaol, inhibit activation of the TLR4 signaling pathway, a finding similar to our predictions [52]. Activation of PI3K signaling protects against cardiomyopathy, which is characterized by adverse remodeling of the heart, diastolic dysfunction, fibrosis, and apoptosis [53]. YAP, a transcriptional regulator protein, is involved in a key pathway in diabetic cardiomyopathy pathogenesis [54]. 6-gingerol is known to protect against hypoxia-induced myocardial injury by activating the PI3K/Akt pathway [55, 56]. Furthermore, it has been reported that 6-gingerol treatment inhibits YAP activation by increasing its phosphorylation and preventing translocation into the nucleus [57]. Although it remains to be determined whether PPARα is a direct molecular target of 6-gingerol, it is known that 6-gingerol activates the glucagon-like peptide-1 mediated insulin secretion pathway [55], which inhibits PPARα-mediated lipid accumulation and toxicity in cardiomyopathy [58]. Additionally, the association between GPR120 and 6-gingerol is also unknown. However, a previous study has reported that in silico docking of gingerol to GPR120 yields negative binding energy, suggesting there is a high likelihood of the compound directly binding to GPR120 [59]. Activation of GPR120 inhibits TAK1 phosphorylation, which is associated with the induction of proinflammatory responses including TNF-α, IL-6, and COX-2 via NF-κB and IKKβ activation. These proinflammatory and fibrosis signaling cascades lead to the development of diabetic nephropathy. Li and colleagues have reported that 6-gingerol suppresses NF-κB and IKKβ activation as well as the production of NF-κB-dependent inflammatory cytokines in vivo [60]. Thus, it is reasonable to hypothesize that GPR120 is a direct molecular target of 6-gingerol for protective effects against diabetic nephropathy. From the results of previous studies mentioned, 6-gingerol is a potent NC that ameliorates T2DM complications. OptNCMiner has correctly predicted the previously known targets of 6-gingerol as well as potential targets that are not yet known, exemplifying its capacity to predict NCs relevant for specific diseases.

### An important recommendation for users of OptNCMiner and room for improvement for better performance of OptNCMiner

OptNCMiner was developed to overcome the limitations faced by existing NC identification methods and to support the effective discovery of novel NCs based on the consideration of multi-target interactions. OptNCMiner was built in an SNN structure to enable learning of hidden structural characteristics that determine chemical-protein interactions. The pair-wise input generation and few-shot learning characteristics of OptNCMiner enable this learning even with small datasets. OptNCMiner generates embedding vectors that place compounds with similar target protein interaction closely in a vector space. Thus, OptNCMiner can be used along with other NC discovery methods such as chemical-chemical interaction prediction [61], toxicity prediction [62], and transcription response prediction [31], to better examine functional NCs from different angles. Furthermore, the structure of OptNCMiner, which compares the similarity of properties of known interactions to predict novel interactions, can be applied to other fields, such as protein-RNA interactions, disease-gene interactions, protein–protein interactions, etc. [63–66].

However, there remains room for improvement for better performance of OptNCMiner. One factor limiting the performance of the program is the complexity of NC biological activity in the human body. Slight differences in physicochemical properties can affect the absorption and distribution of NCs, which alters the interaction between NCs and target proteins. For further development, the physicochemical properties of NCs as well as the cellular location of target proteins should be considered in model input and model training. Although in silico docking has validated predicted false positives as true positives, further validation with in vitro and in vivo studies will verify the accuracy of OptNCMiner. This effort of further validation would also clarify the synergistic or unexpected side-effects of multi-target modulating NCs discovered by OptNCMiner.

One important recommendation for users of our model is the careful selection of target proteins for NC discovery. In the example of T2DM complications, only proteins ameliorating the three complications were considered target proteins. However, to identify NCs that only regulate the desired target protein, in practice, possible off-target proteins may be affected based on background knowledge. OptNCMiner can also be used in conjunction with other programs to support holistic NC discovery, such as programs that predict the absorption and distribution of NCs after ingestion.

## Conclusion

In this study, a novel SNN model called OptNCMiner was built to predict multiple target proteins of NCs. Trained to understand similarities between paired fingerprint vectors, OptNCMiner can predict chemical-protein interactions even for proteins with limited and unbalanced training data. We have demonstrated that OptNCMiner can successfully adapt to training data of various sizes and can predict novel chemical-protein interactions. Furthermore, as a use-case scenario, OptNCMiner was used to predict a natural source and its NCs for the potential treatment of T2DM complications. With a careful selection of target protein candidates, OptNCMiner is a powerful tool to predict novel NCs that modulate specific target proteins to elicit the desired bioactivity.

## Supplementary Information

**Additional file 1:** Supplemental tables.

**Additional file 2:** Excel sheet of in silico docking results of false positives.

**Additional file 3:** Excel sheet of predicted interactions between target proteins and NCs and NC containing natural sources.

## Data Availability

The datasets supporting the conclusions of this article are included within the article, additional files, and GitHub at https://github.com/phytoai/OptNCMiner.

## Abbreviations

AUROC: Area under the receiver operation characteristic
BP: Boiling point
DNN: Deep neural network
GPR120: G protein coupled receptor 120
HL: Henry’s Law constant
KOA: Octanol–air partition coefficient
LogD: PH-dependent lipid-aqueous partition coefficient
LogP: Octanol–water partition coefficient
LR: Logistic Regression
MLP: Multi-layer perceptron
MP: Melting point
NB: Naïve Bayes
NC: Natural compound
NP: Natural product
Nrf-2: Nuclear factor erythroid 2-related factor
PCA: Principle component analysis
PI3K: Phosphoinositide 3-kinase
pKa: Acid dissociation constant
PKCβ: Protein kinase C β
PPARα: Peroxisome proliferator activated receptor α
RT: Retention time
SGLT2: Sodium-glucose cotransporter 2
SNN: Siamese neural network
TLR4: Toll-like receptor 4
VP: Vapor pressure
WS: Water solubility
YAP: Yes-associated protein
