# Supplementary material for: OptNCMiner: a deep learning approach for the discovery of natural compounds modulating disease-specific multi-targets
Source: BMC Bioinformatics. 2022 Jun 7;23:218. doi: 10.1186/s12859-022-04752-5 (PMC9175487; doi:10.1186/s12859-022-04752-5)
Supplement: Supplementary file 1 — Additional file 1: Supplemental tables. [file 12859_2022_4752_MOESM1_ESM.docx]

**Additional file 1:**

**Table S1.** The performance of OptNCMiner learning from a single-label test data and full multi-label data

| **Dataset** | **Recall** | **Accuracy** | **AUROC** |
| --- | --- | --- | --- |
| **Single-label dataset** | 0.805^1^ | 0.682^1^ | 0.738^1^ |
| **Multi-label dataset** | 0.772 | 0.027 | 0.730 |

^1^ Performance metrics for single-labeled dataset were calculated as weighted average of metrics of all proteins composing the dataset.

**Table S2.** Reference validation of false positives from few-shot learning dataset prediction

| **Target Protein** | **Chemical Name** | **Reference Validation** | **In silico docking score** |
| --- | --- | --- | --- |
| ESR | Lemildipine | Hsu et al.  https://doi.org/10.1038/srep06437 | -22.082 |
| MTORC1 | Androstenedione | Boulay et al.  https://doi.org/10.1158/1078-0432.CCR-04-2402 | -16.988 |
|  | Abiraterone Acetate | Floc'h et al.  https://doi.org/10.18632/oncotarget.771 | -22.322 |
| PPARG | Androstenedione | Schoppee et al.  https://doi.org/10.1095/biolreprod66.1.190 | -15.909 |

**Table S3.** Target Proteins for T2DM and available chemical-protein interaction data size

| **T2DM Complications** | **Target Protein (Gene Name)** | **Desired Outcome** | **Actives/Available data size** |
| --- | --- | --- | --- |
| Cardiovascular & Cardiomyopathy | Peroxisome proliferator activated receptor α (PPARα) | Agonist | 2431/2708 |
|  | Yes-associated protein (YAP) | Antagonist | 31/31 |
|  | Phosphoinositide 3-kinase (PI3K) | Agonist | 64/87 |
|  | Protein kinase C β (PKCβ) | Antagonist | 624/746 |
| Diabetic Keratopathy | Toll-like receptor 4 (TLR4) | Antagonist | 45/77 |
| Diabetic Nephropathy | Sodium-glucose cotransporter 2 (SGLT2) | Inhibitors | 1491/1522 |
|  | G protein coupled receptor 120 (GPR120) | Agonist | 506/564 |
|  | Nuclear factor erythroid 2-related factor 2 (Nrf-2) | Agonist | 154/225 |

**Table S4.** Model Performance on T2DM complications scenario

| **Applied method** | **Target Protein** | **Recall** | **Accuracy** | **AUROC** | **Count** |
| --- | --- | --- | --- | --- | --- |
| **Transfer learning** | **GPR120** | 1.000 | 0.973 | 0.985 | 34 |
|  | **Nrf-2** | 0.875 | 0.995 | 0.936 | 8 |
|  | **PKCβ** | 1.000 | 0.993 | 0.996 | 37 |
|  | **PPARα** | 0.991 | 0.863 | 0.853 | 219 |
|  | **SGLT2** | 1.000 | 0.998 | 0.999 | 73 |
|  | **weighted average** | 0.992 | 0.915 | 0.910 | 371 |
| **Few-shot learning** | **PI3K** | 1.000 | 0.813 | 0.850 | 6 |
|  | **TLR4** | 1.000 | 0.438 | 0.591 | 5 |
|  | **YAP** | 1.000 | 0.750 | 0.818 | 5 |
|  | **weighted average** | 1.000 | 0.676 | 0.759 | 16 |
